# Establishing large mammal population trends from heterogeneous count data

**DOI:** 10.1002/ece3.70193

**Published:** 2024-08-22

**Authors:** R. Pradel, P.‐C. Renaud, O. Pays, P. Scholte, J. O. Ogutu, F. Hibert, N. Casajus, F. Mialhe, H. Fritz

**Affiliations:** ^1^ CEFE, Univ Montpellier, CNRS, EPHE, IRD Montpellier France; ^2^ Sustainability Research Unit, Faculty of Science, George Campus Nelson Mandela University George South Africa; ^3^ Cirad, UPR Forêts et Sociétés Montpellier France; ^4^ Forêts et Sociétés, Univ Montpellier, Cirad Montpellier France; ^5^ Univ Angers, BIODIVAG Angers France; ^6^ REHABS International Research Laboratory CNRS‐Université Lyon 1‐Nelson Mandela University, George Campus George South Africa; ^7^ German Development Cooperation (GIZ) Addis Ababa Ethiopia; ^8^ Biostatistics Unit, Institute of Crop Science University of Hohenheim Stuttgart Germany; ^9^ Université de Lyon, Université Lyon 1, CNRS, Laboratoire de Biométrie et Biologie Evolutive UMR 5558 Villeurbanne France; ^10^ FRB‐CESAB Montpellier France; ^11^ Department of Geography, CNRS 5600 EVS University Lumière Lyon 2 Bron France

**Keywords:** Bayesian modeling, heterogeneous wildlife censuses, partial counts, popbayes R package, population rate of increase, population trend, relative growth rate, total counts, wildlife management and conservation

## Abstract

Monitoring population trends is pivotal to effective wildlife conservation and management. However, wildlife managers often face many challenges when analyzing time series of census data due to heterogeneities in sampling methodology, strategy, or frequency. We present a three‐step method for modeling trends from time series of count data obtained through multiple census methods (aerial or ground census and expert estimates). First, we design a heuristic for constructing credible intervals for all types of animal counts including those which come with no precision measure. Then, we define conversion factors for rendering aerial and ground counts comparable and provide values for broad classes of animals from an extant series of parallel aerial and ground censuses. Lastly, we construct a Bayesian model that takes the reconciled counts as input and estimates the relative growth rates between successive dates while accounting for their precisions. Importantly, we bound the rate of increase to account for the demographic potential of a species. We propose a flow chart for constructing credible intervals for various types of animal counts. We provide estimates of conversion factors for 5 broad classes of species. We describe the Bayesian model for calculating trends, annual rates of population increase, and the associated credible intervals. We develop a bespoke R CRAN package, popbayes, for implementing all the calculations that take the raw counts as input. It produces consistent and reliable estimates of population trends and annual rates of increase. Several examples from real populations of large African mammals illustrate the different features of our method. The approach is well‐suited for analyzing population trends for heterogeneous time series and allows a principled use of all the available historical census data. The method is general and flexible and applicable to various other animal species besides African large mammals. It can readily be adapted to test predictions of various hypotheses about drivers of rates of population increase.

## Graphical abstract

Valuable data of wildlife population counts in game reserves has been accumulating over time. Counts are yet often of various natures and collected in a haphazard way. As a consortium of managers and methodologists, we have devised reasonable rules for rendering counts comparable and provide a flexible analytical tool for estimating population trajectories.

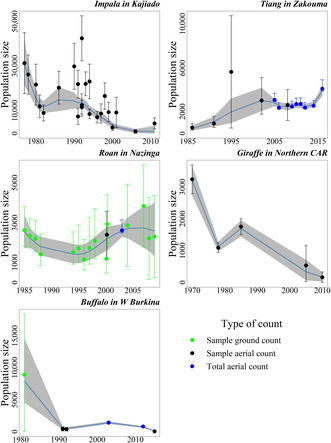

## 1 INTRODUCTION

In the wake of the unfolding, unprecedented biodiversity loss (Ceballos et al., 2015), monitoring wild animals is crucial for building effective conservation and management strategies (Burton, 2012). The monitoring data must be appropriately analyzed to extract reliable insights into trends, rates of increase, changes in trajectories and other population characteristics to inform conservation decisions. Much effort has been devoted to improving data collection and analysis methods (Hammond et al., 2021). Authors often emphasize the necessity of collecting high‐quality data using standardized protocols as a primary requirement (Infantes et al., 2022). Many consider supplementing basic counts with additional information such as mark‐recapture data (Boyd & Punt, 2021) or radio telemetry (Blum et al., 2024).

For analysis, Integrated Population Models (IPM) formulated as state space models (Blum et al., 2024; Mazzetta et al., 2007; Schaub & Kery, 2021) are currently favored (Boyd & Punt, 2021), along with generalized additive models (GAM) (e.g., Forney et al., 2021; Frankel et al., 2022), analyzed in a Bayesian framework (Wood, 2017). Methodologies for analyzing long‐term survey data have been particularly well‐studied in the context of nationwide bird censuses, showing a trend toward more flexibility (Generalized Linear Models (GLM): ter Braak et al., 1994, GAM: Fewster et al., 2000, Hierarchical models: smoothed hierarchical model: Amano et al., 2012).

At the data collection stage, many factors may hamper visibility, such as vegetation cover, topography (Blum et al., 2024), animal behavior, group size, and observer experience (Bristow et al., 2019). Studies in the United States have examined how the undercounting by aerial surveys of large mammals (bighorn *Ovis canadensis nelsoni*, Blum et al., 2024; Elk *Cervus canadensis*, Bristow et al., 2019; feral burro *Equus asinus*, Hennig & Schoenecker, 2023; bisons *Bison bison*, Terletzky & Koons, 2016) could be corrected. But the proposed solutions are generally too expensive to implement on a wide scale or routinely.

Unlike research programs, monitoring programs of protected areas or populations (Arciszewski et al., 2023) often operate with limited funding and must accommodate data typically characterized by heterogeneities in sampling techniques, effort, or frequency. This situation is typically encountered in low‐income countries, but not exclusively. For example, wildlife agencies in North America face constraints when monitoring and managing wildlife (e.g., Caughlan & Oakley, 2001; Sands & Pope, 2010); which is especially true for many Tribal nations (Shamon et al., 2022).

Therefore, if estimating true population size is particularly elusive in this context, determining whether the population is decreasing, increasing, or stable should become the more reasonable target. Appropriate analytical approaches would therefore require reexamining existing methods for estimating population trends, which often demand homogeneous and larger datasets than typically available, besides statistical skills that often transcend those available to most conservation management teams. This deficiency can be partly alleviated by developing accessible off‐the‐shelf software packages.

In this paper, we aim to address census data heterogeneity, paucity of data sources, and user‐friendly methods for estimating wildlife trends. This work stems from a collaborative project aiming at collating archived information to assess the status and trends of large herbivores in West and Central Africa. Census data from protected areas in these subregions of the continent typify the preceding challenges. The method and associated software package we present here are thus directly motivated by concrete challenges encountered while analyzing trends for these heterogeneous wildlife census data.

Analyzing population trends using multi‐taxa wildlife censuses faces many additional challenges. The first concerns accommodating unequal intervals between consecutive censuses in trend models. Very few protected areas have long‐term monitoring programs that provide regular wildlife abundance estimates typically because of budgetary constraints. However, modeling trends for irregularly spaced time series of censuses is challenging. Gaps in time series can be mitigated when covariates are available at times where counts are missing (Fewster et al., 2000). Unfortunately, such covariates are rarely available in most population time series (Humbert et al., 2009). Smoothing techniques, such as log‐linear Poisson regression (Fewster et al., 2000), dynamic GLM (Mazzetta et al., 2007), and hierarchical models (Amano et al., 2012), that is assuming some regularity in the way the population is changing in the intervals between available counts, remains an option.

The second challenge relates to accommodating data obtained with different census techniques and sampling strategies with varying accuracies in the same trend model. A comprehensive review of census techniques can be found, for example, in Sutherland (2006). As methodologies for counting wildlife have been increasingly improved (Borchers et al., 2002), census techniques, sampling strategies, and the accuracy of counts have strongly evolved through time. This evolution complicates integrating new and old wildlife counts, especially those obtained 5–6 decades earlier, in the same trend model. Old counts, although usually less accurate, are nevertheless crucial, as they often provide the only information on wildlife population status at the early stage of establishment for most protected areas. The earlier estimates are sometimes provided by managers based merely on their expertise. Yet, resorting to expertise is increasingly recognized as a valid practice in conservation (Kuhnert et al., 2010) and practitioners' expert knowledge and expertise are considered especially valuable (Drescher et al., 2013).

In practice, due to cost constraints (Gaidet‐Drapier et al., 2006), counting protocols are limited to four categories: whether they are census or sampling, that is total or partial counts, and carried out by air or ground. Additionally, changes in governance often lead to changes in methodology. Detection rate is a key component of the accuracy of abundance estimates (Jachmann, 2002; Ridpath et al., 1983; Van Hensbergen & White, 1995, for some examples) and differs between observations from the air or from the ground. Beyond vegetation cover, the main factors influencing the detectability of wild mammals are body size and color (Jachmann, 2002). Therefore, when using multiple census techniques, an estimation bias is typically introduced as aerial counts usually give fairly accurate estimates for large, dark‐bodied animals but usually underestimate small, light‐bodied ones (Jachmann, 2002). Conversely, ground techniques tend to be less accurate for large‐bodied and highly mobile animals.

As no standardized conversion factors have been set for these known biases, practitioners tend to use only a subset of the available data obtained with the same census technique (see Redfern et al., 2002 for an attempt at correcting the probability detection bias on aerial counts). However, for some protected areas, ignoring some counts can considerably limit time series analysis and create gaps between counts. Unfortunately, methodologies for integrating heterogeneous wildlife censuses and modeling population trends are still crude (Humbert et al., 2009). Such methodologies need improvement to better suit practitioners' needs and enable reliable decision‐making. In the absence of appropriate methods, standard statistical tools like linear regression are often used even though they are inappropriate for handling irregularly spaced counts, or variation in detectability among species due to body size, color, and census techniques (Krebs, 2006). Standard methods also do not impose an upper bound on population growth between successive counts implied by the demographic potential of surveyed species (Fisher, 1930).

Not surprisingly, most reports or publications use crude methods by simply comparing the first and last counts and calculating a percentage change in population size (see Barnes et al., 2016; Renaud, 2005; Renaud et al., 2006; Stalmans et al., 2019 for some recent examples in scientific papers or expert reports). More elaborate attempts fit linear trends (Bart et al., 2003) or calculate an index of population change based on the first count in the time series (Barnes et al., 2016; Craigie et al., 2010; Tolimieri et al., 2017 for some examples). Therefore, many contemporary time series analyses of wildlife censuses limit decision‐making by simply deriving an index of change from the first available count, thereby ignoring the absolute amount and timing of changes (see Craigie et al., 2010 for some examples).

Therefore, we require analytical approaches able to incorporate salient features of wildlife populations and make the best use of all available count data. In particular, such approaches should consider the precision of each count and give more weight to the more precise counts in the series. They should also consider the demographic potential, usually measured by the “maximum instantaneous rate” of increase (Sinclair, 2003) or maximum relative growth rate, *r*_max_. Values for *r*_max_ can be found in the literature for a number of species (see Table 3).

Many models analyzing population count series have at their core a population growth model (e.g., Dennis et al., 2006; Forney et al., 2021; Hostetler & Chandler, 2015; Kidwai et al., 2019; Yeiser et al., 2018), such as exponential, Ricker or Gompertz. Some of these models include *r*_max_ among their parameters. However, the role of *r*_max_ in these models differs from ours. These models make strict assumptions about the form of population growth and use data to estimate model parameters including *r*_max_. In contrast, we do not make assumptions about the form of population growth and see *r*_max_ as external information useful for evaluating trends more realistically.

The relative growth rate, *r*, is related to the percentage change in population size per time unit *R* by *r* = ln(1 + *R*), making it, in our opinion, the appropriate quantity to model. The relative growth rate also has the advantage of being chiefly influenced by prevailing conditions (climate, food resources, predation, competition, governance). Thus, if these conditions change substantially and progressively, then the relative growth rate may also change. Incorporating a positive dependence between successive values of the relative growth rate is thus a natural way of smoothing the population trajectory. Ultimately, the choice of how to model r can open up the way to include environmental variables, and thus to the possibility of testing potential functional relationships between environmental and demographic changes.

Here, we present a Bayesian approach for estimating population trajectories from heterogeneous counts, including potentially large time gaps (>10 years in some examples in Section 3.3), and illustrate its application using several populations of large mammals from African protected areas. This approach is programmed in a bespoke R package called “popbayes” (Casajus & Pradel, 2023), which includes routines for preprocessing counts and carrying out the proposed Bayesian analysis. Preprocessing is required because certain old censuses often lack the minimum information needed for statistical analysis, such as a measure of precision, or are not directly comparable among themselves due to changes in census methods over time.

**FIGURE 1:**
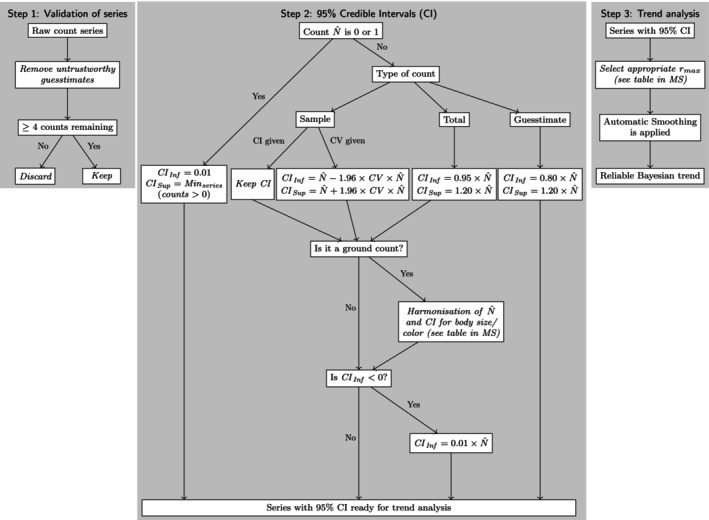
Flowchart describing the process for conducting a trend analysis for a heterogeneous data set of wildlife counts.

We start by presenting the diverse types of data sets for which this type of analysis can be performed, highlighting the challenges they present and proposing how to overcome them and extract as much information as possible. We then derive conversion factors for harmonizing parallel pairs of aerial and ground counts. We advocate the use of 95% credible interval as a common measure of precision for all counts in a series and propose ways to construct it when no measure of precision is available (step 2 of Figure 1). Finally, the model accounts for the demographic potential of a species, expressed as its intrinsic population growth rate (Sibly & Hone, 2002). All the steps in the method are summarized in a flow chart (Figure 1).

We then illustrate the steps in data preparation and processing with an example, showing how original raw counts are used to obtain the final population trajectory. The robustness of our procedure is illustrated through several examples with increasing levels of managerial and analytical challenges. These examples cover a broad spectrum of real census data, ranging from relatively rich, method‐homogeneous count series with changes in counts incompatible with the demographic potential of the species, to sparse and highly method‐heterogeneous series (Table 1). Finally, we illustrate potential interpretations of the modeled trajectories, underscoring the central role of the relative growth rate.

**TABLE 1:** Summary of the analytical and managerial challenges associated with the time series we considered in Section 3.2.

Site	Species	Origin	Analytical and managerial challenges
Kajiado County (Kenya)	Impala (*Aepyceros melampus*)	Joseph Ogutu	All counts are aerial samples but some very heterogeneous population size estimates at close dates
Zakouma National Park (Chad)	Tiang (*Damaliscus lunatus tiang*)	Collated by us (multiple sources)	All counts aerial but a shift from sampling to total counts occurred in the middle of the period
Nazinga Game Ranch (Burkina Faso)	Roan (*Hippotragus equinus*)	Collated by us (multiple sources)	Mainly sample ground counts with occasional changes in both sampling and field methods
Bamingui‐Bangoran and Monovo Gounda Saint Floris National Parks complex (Central African Republic)	Giraffe (*Giraffa camelopardalis antiquorum*)	Collated by us (multiple sources)	Method consistent but very few counts spread apart
W National Park (Burkina Faso)	Buffalo (*Syncerus caffer brachyceros*)	Collated by us (multiple sources)	Both few counts and high heterogeneity of methods

## 2 MATERIALS AND METHODS

### 2.1 The data sets and their challenges

**FIGURE B1:**
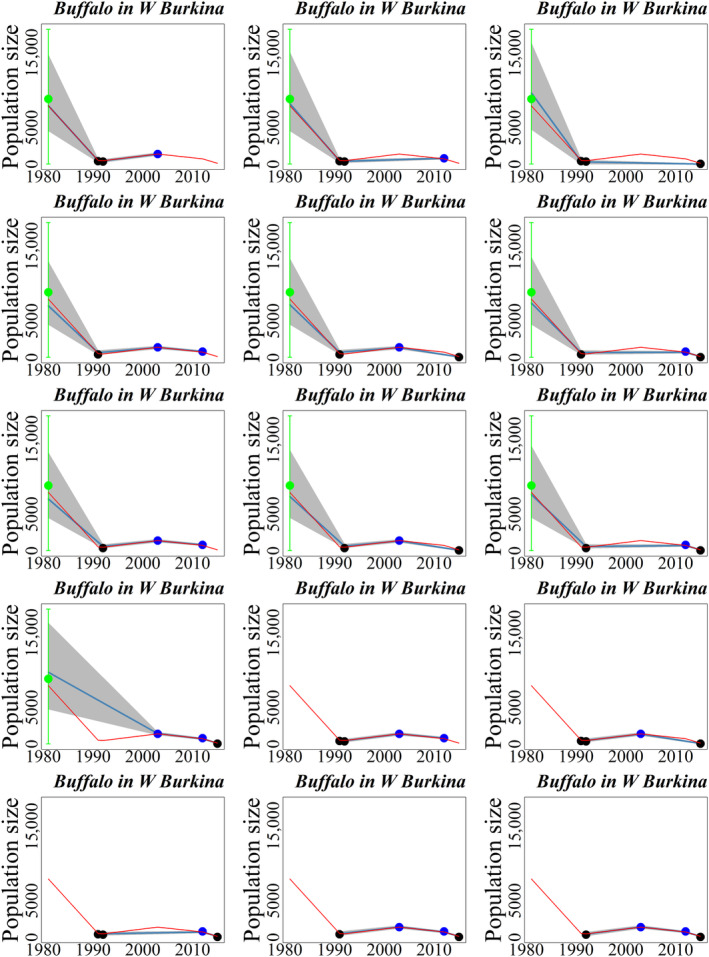
Analyses of all possible subseries of 4 counts out of the 6 counts available for the Buffalo at W Burkina are presented. In each subpanel, the red curve represents the results using all 6 counts (see Section 3.2), while the blue shows the results with the 4 retained counts. There is minimal difference for the overlapping parts. The main loss is the total lack of information when the series is shortened at the beginning or the end. Missing an intermediate point can obscure the timing of changes. For instance, the population drop between 1980 and 2000 occurred entirely before 1990, but this detail is lost when the two 1990 counts are missing (subfigure 4,1). Dot colors are as shown in Figure 3.

A database of census data of large herbivores in several African protected areas was created during an earlier project, called Afrobiodrivers (https://www.fondationbiodiversite.fr/en/the‐frb‐in‐action/programs‐and‐projects/le‐cesab/afrobiodrivers/). The protected areas then considered are scattered across West and Central Africa where data on wildlife populations are often scarce and heterogeneous. Data were digitized from paper archives. In this article, we use a subset of the Afrobiodrivers collated data supplemented with data from East and Southern Africa from published references or accessible sources. To support inference on population trends, a data set must comprise a minimum number of counts; we settled on 4 as a rule of thumb. While a count series of 3 or less will not be accepted by our code, a more stringent rule may of course be adopted by the user. We illustrate the effect of the 4‐count rule by subsampling a data set (see Figure B1 of Appendix B). All counts in this paper are aerial or ground counts, although expert guesstimates could have been used as well. Each eligible count was associated with its date (i.e., the year), site (e.g., protected area), species, and counting method separated into field and statistical methods (Eikelboom et al., 2019). The field method refers to the counting technique (aerial or ground count) whereas the statistical method refers to the sampling strategy (total or partial counts); expert estimates would be treated as a third statistical method. If a measure of precision (e.g., confidence interval) was available, it was retrieved together with the count. We treat the case where no measure of precision is provided in Section 2.3.

**FIGURE B2:**
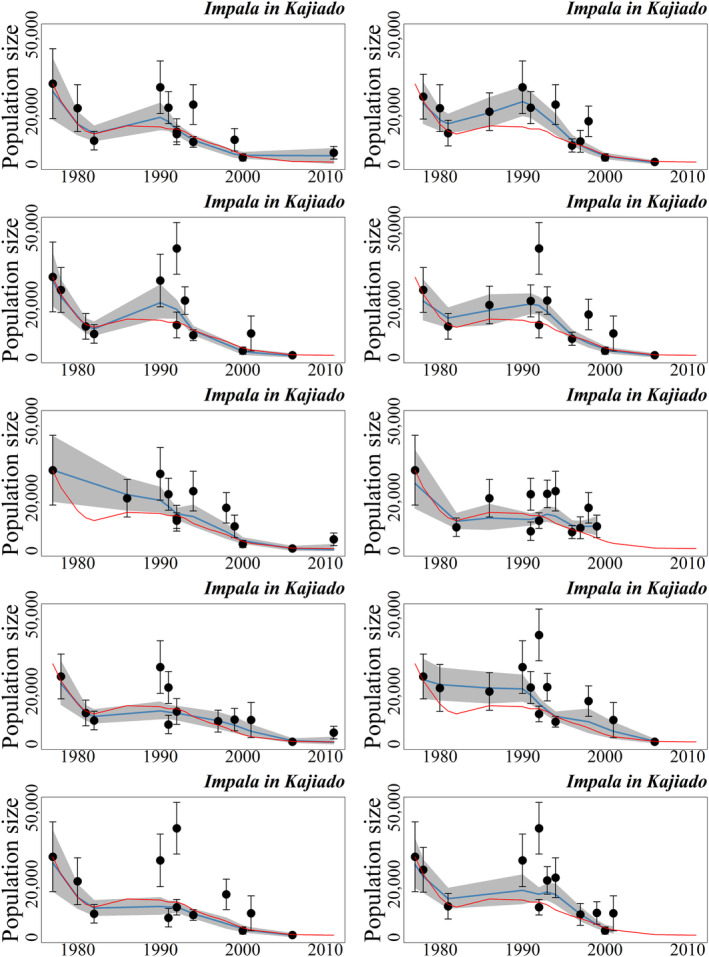
To further illustrate the robustness of the method, we present the results from a significantly reduced data set (<50% counts retained). We analyze 10 random subsamples of 12 points (out of 25) from the impala count series at Kajiado analyzed in Section 3.2 and Figure 3. The results from the complete data set are represented by the red curve. The general pattern remains consistent: An initial decrease prior to 1980, followed by stabilization or a slow recovery during the 1980s, and a new decline starting in the early 1990s. However, the absolute numbers are less reliable.

It has long been known that counting from the air or from the ground yields different estimates and that the discrepancy varies with the species, notably its size and color (Greene et al., 2017; Jachmann, 2002). We adopt the approach of seeking to estimate a conversion factor between ground and aerial counts as in Greene et al. (2017), assuming this factor depends primarily on a species' characteristics (but see Section 4). However, as our primary interest is in the estimation of population trend, we do not assume like Redfern et al. (2002) that one method provides true population size (Figure B2).

Hence, if the expected count by method A (aerial) is C_A_, and the expected count by method G (ground) is C_G_, C_A_ = β C_G_. Knowing β allows us to calculate what the count would have been if aerial counting had been used instead of the ground counting. To estimate the conversion factor β thus defined, we used 166 partial counts (83 pairs) carried out from the ground and from the air almost at the same time (at most within a month). The partial counts come from four protected areas, Hwange National Park, Nazinga Game Ranch, Maasai Mara National Park, Lupande Game Management Area, mainly hosting wooded savannas (data from Cornélis, 2000 for Nazinga, J. O. Ogutu for Mara, H. Fritz for Hwange, Jachmann 2002 for Zambia).

As detection probability is influenced by species size and color (e.g., East, 1999), we relied on the expert knowledge of our team members with protected area management experience to define broad categories of species likely to share a similar bias. We retained five species classes that can be used across sites: (1) elephant *Loxodonta africana*; (2) giraffe; (3) large dark species (buffalo and sable); (4) large light and brown species with female body weight above 150 kg (e.g., eland *Tragelaphus derbianus*, kudu *Tragelaphus strepsiceros*, Lichtenstein hartebeest *Alcelaphus buselaphus lichtensteinii*, roan, waterbuck *Kobus ellipsiprymnus*, blue wildebeest *Connochaetes taurinus taurinus*, plain zebra *Equus quagga*); (5) medium light and brown species with adult female body weight above 10 kg but below 150 kg (e.g., gazelles *Gazella* spp., impala, kob *Kobus kob kob*, red hartebeest *Alcelaphus buselaphus caama*, topi *Damaliscus lunatus topi*, warthog *Phacochoerus africanus*). While these species are typically found in habitats suitable for ground and aerial surveys, the detection of smaller species, for example, duikers *Cephalophus* spp., dik‐dik *Madoqua* spp. or even oribi *Ourebia ourebi*, by standard aerial surveys, in well‐vegetated savanna landscape, is typically too variable and unreliable to analyze.

The conversion factors were computed from the pairs of parallel counts using a Bayesian model that gives more weight to the more precise estimates (see Appendix A for details). The results are consistent with expectation, namely a higher count estimate for medium and large, light brown species from the ground, and a higher estimate from the air for elephants and dark species, sable and buffalo. Using these conversion factors allowed us to establish population trends with mixed census methods. This implies, for instance, that the estimated number of elephants based on ground counts would have to be adjusted by a multiplicative factor to be comparable with an aerial count estimate obtained on another date (Table 2). We come back to this point in the Discussion section.

**TABLE 2:** Multiplicative conversion factor to apply to an aerial count to obtain an equivalent ground count. The data used are available from the authors upon request.

Species class based on color and/or body mass^a^	Conversion factor [95% CI]
Medium‐sized light and brown species (20–150 kg)	6.747 [6.701, 6.792]
Large light and brown species (>150 kg)	2.302 [2.244, 2.359]
Large dark species (>150 kg)	0.561 [0.545, 0.577]
Giraffe	3.011 [2.936, 3.083]
Elephant	0.659 [0.657, 0.662]

^a^ Medium‐sized light and brown species include impala and tiang, large light and brown species, roan, blue wildebeest and eland, and large dark species, buffalo.

### 2.2 Associating a confidence interval to each individual count

A measure of precision is generally provided along with the counts in the literature, but not always. This measure may be a standard error, a variance, a coefficient of variation, or a confidence interval (CI). If the distribution is specified, a 95% CI can always be derived. If the distribution is not specified, we assume a normal distribution, as other distributions would likely be specified if used. Hence, we decided to use the 95% CI as our standard measure of precision.

Sometimes, the derivation of a CI leads to nonsensical results, such as a negative lower bound. Since negative values for counts are illogical and can cause problems in calculating trajectories (see below), we replaced any negative lower bound with 0.01. When a species becomes locally extinct during monitoring, confidence intervals should be [0,0], which is not acceptable for the algorithm. In such cases, we set the CI at [0,0.01].

A measure of precision is often lacking when counts are reported as total counts or expert guesstimates. Several of our team members with extensive practical experience in actual wildlife censuses and their use agreed that a reasonable rule for expert guesstimates of large savanna mammals is that the true population size would be within 20% more or less than the expert guesstimate in 95% of cases. For total counts, it is much less likely that the true population size is lower than the count, as this can only occur when some individuals are double‐counted. Hence, we use asymmetric 95% CIs. The lower bound is set at 5% less than the count; while the upper bound remains 20% above the count.

### 2.3 Inferring population trajectory from counts

In modeling population trajectory, the basic parameter is the relative growth rate, defined as:

*r* = ln(*N*_*t* + 1_/*N*_*t*_) where *N*_*t*_ is the population size at time *t*. The default unit of time is the year.

Although changes in effective environmental conditions may sometimes be abrupt, most of the time, neighboring years tend to resemble each other. We therefore implemented a constraint between successive relative growth rates as follows:

$$r_{t+1}\sim N\;\left(r_{t},0.01\right)$$

This means that *r*_*t* + 1_ is drawn from a normal distribution with a mean *r*_*t*_ and a standard deviation of 0.1. This forms the smoothing part of the algorithm. The reciprocal of the variance, called precision in statistics, thus has a default value of 100. Reducing this value would produce a rougher curve (see Section 3.1).

The first relative growth rate *r*_1_ is drawn from a very liberal distribution with a 0 mean and unit variance, corresponding to no change in population size.

$$r_{1}\sim N\;\left(0,1\right)$$

The first population size $N_{1}$ is drawn from a uniform distribution between half and double the first count:

$$N_{1}\sim \text{Unif}\left(C_{1}/2,2^{*}\;C_{1}\right)$$

Additionally, if immigration can be ignored, the demographic potential of a species caps its relative growth rate (see Table 3). Whenever a higher value is drawn for *r*, it will be replaced by *r*_max_:

$$r\leq r_{\max}$$

**TABLE 3:** The intrinsic rate of increase (*r*_max_) of the population was assessed from the body mass of adult females in the studied species.

Species	Body mass (W) of adult female (kg)	*r*_max_	Reference (see footnote as well)
Impala	55	0.401^a^	Kingdon and Hoffmann (2013)^c^
Tiang	127	0.299^a^	Child et al. (1972)^c^ adjusted from Sachs (1967)
Blue wildebeest	230	0.247^a^	Kingdon and Hoffmann (2013)^c^
Roan	250	0.242^a^	Kingdon and Hoffmann (2013)^c^
Buffalo	400	0.208^a^	Cornélis et al. (2014)^c^
Eland	450	0.150^b^	Sinclair (1996)^b^
Giraffe	702	0.175^b^	Suraud et al. (2012)^b^
Elephant	2873	0.112^b^	Foley and Faust (2010)^b^

*Note*: *r*_max_ is assessed using 1.375 W^−0.315^ from Sinclair (1996) in *a*, and reported from the literature when a demographic analysis had been conducted in *b*. Reference for body mass is indicated in *c*.

The second part of the model describes the observational process. It uses the provided confidence intervals as two series of counts corresponding to the lower (Cmin) and upper (Cmax) bounds of the 95% CIs. From each Cmin–Cmax pair, we derive a standard deviation assuming a normal distribution:

$$\sigma =\left(\text{Cmax}–\text{Cmin}\right)/3.93$$

In turn, this standard deviation is used as the standard deviation of the normal distribution of the count around the unknown population size (*N*).

$$C\sim N\left(N,\sigma^{2}\right)$$

The model is implemented in a Bayesian framework using the program Jags (Plummer, 2003). The Jags code for the model is given in Appendix A. A flow chart summarizes all the above steps (Figure 1).

## 3 RESULTS

### 3.1 An example of how to transform raw data into a population trajectory: Roan antelope (*Hippotragus equinus*) in Nazinga game ranch (Burkina Faso)

**FIGURE 2:**
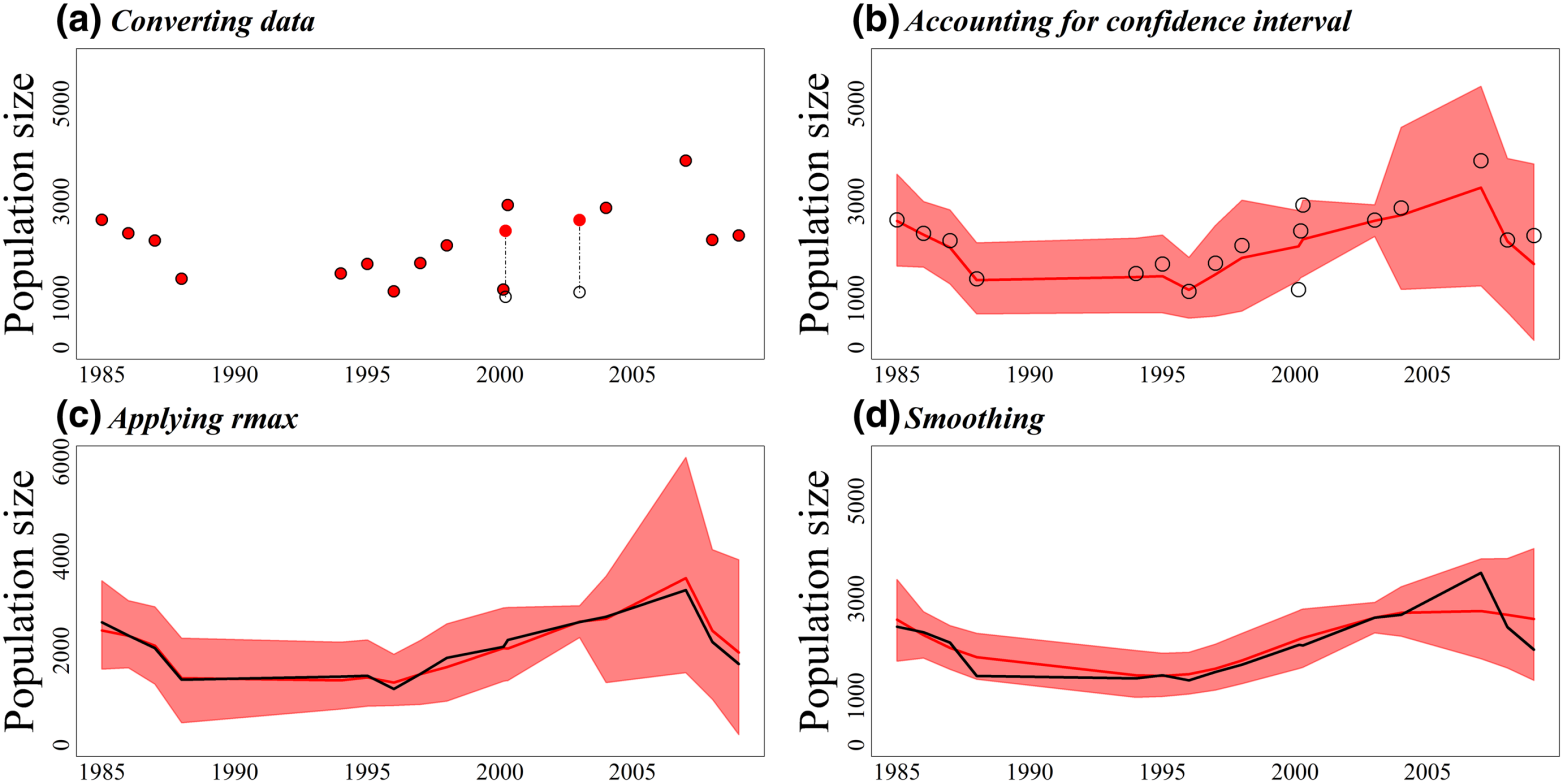
Successive steps in modeling counts of roan antelope in Nazinga: Each panel compares the preceding (black) to the next (red) situation: (a) from raw (open black circles) to converted data (full red circles) according to the field method used; (b) from converted data (open black circles) to a fitted curve accounting for the count precisions (red curve); (c) curve adjusted to account for demographic potential (*r*_max_); (d) smoothing by assuming that the relative growth rate *r* changes progressively.

In Nazinga, the predominant field method has been ground censuses. A total of 18 censuses were conducted between 1985 and 2009, two of which were aerial (in 2000 and 2003). Because, according to experts, ground counts are deemed more reliable for roan antelopes, the two aerial counts are first transformed to render them comparable to the ground counts (Figure 2a). Given that roan antelopes are large brown species, the conversion was achieved by multiplying the aerial counts by 2.302 (Table 2). After this conversion, the two aerial counts align more closely with the general trend, although the ground count from February 2000 appears somewhat outlying.

An interesting feature of this data set is that 3 counts were carried out in 2000: a sample ground count by car in February, a sample aerial count in March, and a sample ground count by foot in April. The method can handle different counts obtained in the same year, but it is also possible to use fractional years. We opted for the latter option because it better matches the data. When accounting for count precisions, the curve excludes the count for February 2000 (Figure 2b), which lies outside the 95% credible envelope. A high rate of increase is observed for a very brief period in 2000. When the maximum relative growth rate *r*_max_ is factored in, this sharp increase disappears (Figure 2c).

However, assuming no sudden year‐to‐year changes during the period leads to the smoother curve in Figure 2d. This is achieved by changing the smoothing precision (see Section 2.3) from a very low value of 1 (Figure 2b,c) to 100. This last value of the smoothing precision and the maximum relative growth rate, which are the default options in the package popbayes, are used in the remainder of the paper.

### 3.2 Robustness of the approach to heterogeneity in field and statistical count methods

**FIGURE 3:**
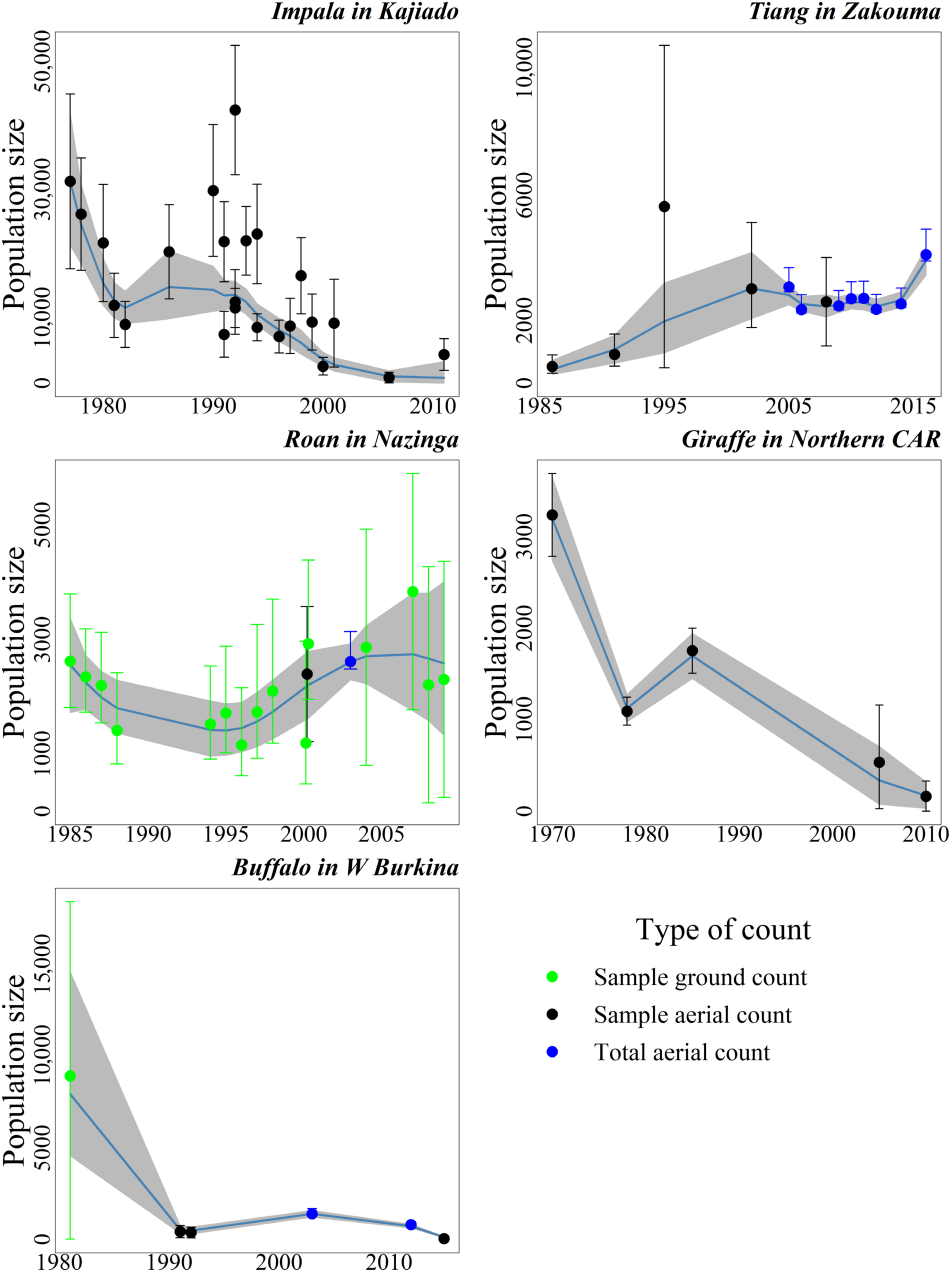
Deriving trajectories for very heterogeneous wildlife survey data sets: An illustration with five case studies portraying various analytical and managerial challenges (see Table 1). The gray areas represent the 95% credible band.

This section presents examples that showcase the model's ability to deal with increasing challenges. The counts of impalas in Kajiado (Figure 3a) represent an ideal situation: a single method (aerial sampling) has been used consistently for 23 aerial counts carried over 33 years (between 1977 and 2011) and all counts come with an associated precision (95% confidence interval). Yet, these counts are highly variable, even for close dates. For instance, a count of 6345 (before conversion) in 1992 is surrounded by two counts of 1886 and 1747 in the same year. Conflicting counts are also found in 1991 (2 counts) and 1994 (2 counts).

Here, unlike with the roan antelope in Nazinga, we have kept the exact same date for the counts conducted in the same year for illustration. The relatively higher precisions and the accumulated evidence of the two consistent low counts of 1992 heavily influence the curve, drawing it away from the high count of the same year. In 1994, information from the following years mainly draws the curve toward the lower count. Overall, the model captures the temporal trend well, avoiding unrealistic ups and downs. The precision indicated by the credible envelope is better for periods with numerous counts.

The tiang counts in Zakouma exemplify a change of methodology. All counts are aerial, but before 2005, they were sample counts; afterwards, except for 2008, they were total counts. Total counts are considered reliable, particularly regarding the minimum number of individuals in an area: a 95% confidence interval is built with a lower limit of 5% below the actual count and an upper limit of 20% above. Here, we have a high count of 2450 in 1995 that follows a previously low count of 400 in 1991 and precedes a count of 1310 in 2002. However, the precision of this point is low. Moreover, the species' demographic potential limits the multiplication of the population size over 4 years by a factor of 3.32. Despite the degraded precision from 1991 to 2002, the model avoids the high 1995 count (estimate for this year is 856) and fits a more reasonable temporal trend (Figure 3b). The Nazinga roan count series has 2 aerial counts interspersed among 17 ground counts, requiring the ground counts to be rescaled to be comparable with the aerial counts. The large imprecision of the ground counts is conspicuous in 2000 when two counts were carried out, yielding point estimates of 2929 and 1192, respectively (before rescaling). The aerial count of 2003 is very influential as it is a total count. The model predicts a population low in 1995, with the subsequent upward trend limited by the species' demographic potential (Figure 3).

Some series have very few points. For example, only five giraffe counts are available for the northern Central African Republic from 1970 to 2010. However, the sampling method has consistently been aerial surveys. The model closely follows the counts, which are too far apart to influence each other, except for the last two counts, where the more precise last count draws the curve below the less precise second last one (Figure 3d). The precision expressed by the credible envelope suggests a phase of population increase in the late 1970s and early 1980s, corresponding to a lapse in the continuous decline. The buffalo counts in W Burkina exemplify high heterogeneity with a mixture of field and statistical methods. They are also few and widely spaced (Figure 3e). After converting the only ground count to its equivalent aerial count (the preferred method for this species) and constructing confidence intervals for the two total counts, it appears that this population collapsed during the 1980s, which corresponds to the last episode of rinderpest (Agriculture Ministers' Conference, 2010; Tounkara et al., 2017) before the disease was definitively eradicated from the region by the end of the 1980s (see for instance Kouba, 2013). The later years suggest a modest recovery toward the end of 2000 followed by another dramatic decrease. Again, the counts are too far apart for a refined interpretation, but the precision is sufficient to portray the successive tendencies.

### 3.3 Interpreting trajectories in terms of the relative growth rate

**FIGURE 4:**
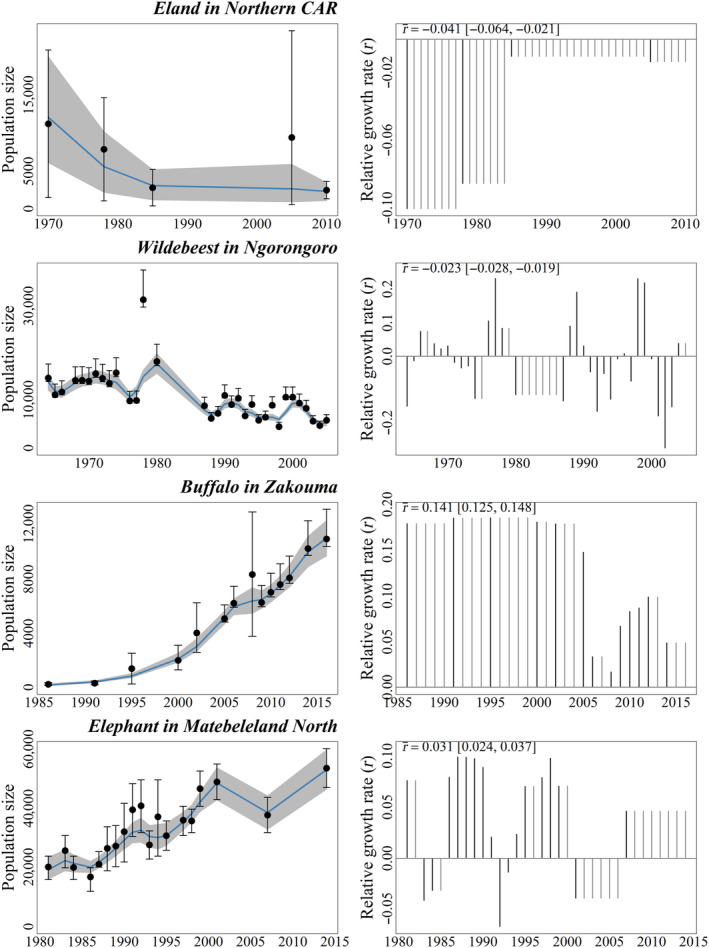
Illustrations of the relative growth rate: the mean *r* over the study period indicates the overall trend while the yearly rate of increase may help identify periods with an opposite trend.

In addition to modeling the trend, the method estimates the relative growth rate (*r*). This provides complementary insights into the population trend as it highlights periods that may deviate from the overall trend. For instance, the mean *r* is negative for both the eland in Northern CAR (1970–2010) and the Wildebeest in Ngorongoro (1964–2005) (Figure 4). However, while the annual *r* series is consistently negative for the eland over the entire survey period, the wildebeest population size increased during 5 intermediate periods (1966–1971, 1977–1980, 1988–1991, 1998–2000, 2004–2005). Similarly, whereas the mean *r* over the entire period is positive for both the buffalo in Zakouma (1986–2016) and the elephant in Matebeleland North (1981–2014), the buffalo population size increased continuously, while the elephant population decreased on two occasions, between 1983 and 1986, and again between 2001 and 2007 (Figure 4).

## 4 DISCUSSION

In our methodological approach, we first established criteria for including data from different census methods to minimize the loss of historical information for a given protected area. We then developed a general and flexible model for modeling trends in heterogeneous wildlife counts and estimating associated uncertainties. To draw realistic population trends, we accounted for the demographic potential of a species. We illustrated the model using data from west, central, and eastern Africa. The trends we estimated are consistent with known historical events in these regions. For instance, the dramatic drop in Burkina Faso's W National Park buffalo population during the 1980s corresponds to a severe episode of rinderpest, to which buffalo is particularly susceptible. Similarly, the documented dramatic decline of giraffe's and eland's populations in Northern Central African Republic during the 1970s align with our findings (see for instance Bouché et al., 2009; Scholte et al., 2022). We also verified that the results remained consistent when only a subsample of the time series was used (see Figure  B1 and B2 of Appendix B), demonstrating that the method is robust and relies on the entire set of counts rather than individual data points. In the following sections, we emphasize the novelty, benefits, and limitations of the proposed method and associated package, popbayes.

The maximum annual population growth rate *r*_max_ is a critical parameter for population management models (Hone et al., 2010). Even though it is rarely reached, it indicates a population's potential to bounce back after a perturbation. Integrating *r*_max_ into trend modeling is therefore crucial to ensure realistic prediction of population recovery. Surprisingly, this has not been commonly done when analyzing real data. Models that focus on population growth often include trend models, but they typically do not set a cap on *r*_max_ (see for instance Dennis et al., 2006; Forney et al., 2021; Hostetler & Chandler, 2015; Kidwai et al., 2019; Yeiser et al., 2018). The use of *r*_max_ is optional in our approach and should be omitted if immigration is suspected. We plan to allow *r*_max_ to be used at specific dates in future versions of the model.

Identifying the determinants of a population's rate of increase using field data is central to gaining a better understanding and managing wildlife populations (Sibly & Hone, 2002). Therefore, combining the analysis of trends in population numbers (often used to assess critical conservation status, e.g., IUCN red listing) with trends in growth rates, provides a much better indicator of whether management, social, or ecological conditions are conducive to population growth. The average rate of increase, we suggest and calculate in the “popbayes” package, effectively quantifies overall trends because over long periods, populations fluctuating naturally in their environment are expected to have an average rate of increase close to 0 (Hone, 1999).

For biodiversity conservationists and managers, the added value of the “popbayes” package lies in its flexibility in building population trends from highly heterogeneous datasets. The tool offers built‐in solutions for integrating wildlife population estimates from different census techniques, allowing users to make full use of all the available data types for an area. It accounts for biases in census methods by considering species' body size and color and managers' expertise on the most appropriate techniques for particular species. The applied conversion factors enable the use of many censuses to model population trajectories. These proposed factors may be substituted by more appropriate values if known. Furthermore, the package allows the use of expert guesstimates. If a guesstimate is deemed reliable, the user can specify its nature, and the package will automatically treat it as such, constructing the default confidence interval as shown in Figure 1. For many protected areas, such expert guesstimates are the only available data on the wildlife population status in the early stages of their official gazettement. Experienced managers are likely to have a thorough understanding or accurate information on species' traits (weight, color) and appropriate census methods for their specific areas. The package allows changes in the reference tables used to calculate conversion factors and *r*_max_ values. The flow diagram may need to be modified accordingly, in particular, if ground methods become the standard reference for a particular area.

The use of the annual population growth rate as an output of the model is a critical asset of the package. Following Hone et al.'s (2010) insights, calculating the annual population growth rate *r* opens the door to a wide range of interpretative perspectives for managers. While many previous approaches have centered their models on log population size (Amano et al., 2012; Fewster et al., 2000; ter Braak et al., 1994), we believe that the population growth rate *r* is a more natural parameter because it directly measures current population dynamics. Hence, the relationship between successive *r* values provides a natural way of smoothing, reflecting the continuity of environmental conditions in successive years. Conversely, a lack of continuity would indicate a dramatic change in conditions. Currently, the package does not allow changes in the degree of smoothing over the time series, but this is a feature we are considering for future versions.

The “popbayes” model is built primarily to maximize the output from heterogeneous time series of count data to facilitate and enhance their use in conservation management. Although the package was motivated by counts of African large mammals, it is general and can be used in other contexts. For example, counts of marine mammals share many similarities (Hammond et al., 2021). More broadly, the package can be applied to monitoring data characterized by varying methods, inconsistent effort levels or frequencies. The package is flexible and can readily be extended to investigate putative drivers of population change. If environmental covariates are available, regression models can be used to link population changes to their putative drivers. Comparison of trends for the same species across different protected areas or of different species in the same area can also be easily implemented. Additionally, if a known perturbation causes a sudden change in environmental conditions, the model can allow for a corresponding sudden change in *r*.

## AUTHOR CONTRIBUTIONS

**R. Pradel:** Conceptualization (equal); formal analysis (equal); methodology (lead); software (equal); validation (equal); visualization (equal); writing – original draft (lead); writing – review and editing (lead). **P.‐C. Renaud:** Conceptualization (equal); data curation (lead); funding acquisition (supporting); validation (equal); writing – original draft (supporting); writing – review and editing (supporting). **O. Pays:** Formal analysis (equal); validation (equal); visualization (equal); writing – original draft (supporting). **P. Scholte:** Data curation (supporting); validation (equal); writing – original draft (supporting). **J. O. Ogutu:** Data curation (supporting); validation (equal); writing – original draft (supporting); writing – review and editing (supporting). **F. Hibert:** Data curation (supporting); validation (equal). **N. Casajus:** Software (equal); validation (equal); visualization (equal). **F. Mialhe:** Validation (equal); writing – original draft (supporting). **H. Fritz:** Conceptualization (equal); data curation (supporting); formal analysis (equal); funding acquisition (lead); validation (equal); writing – original draft (supporting); writing – review and editing (supporting).

## CONFLICT OF INTEREST STATEMENT

Authors have no conflict of interest to declare.

## Figures and Tables

**FIGURE A1:**
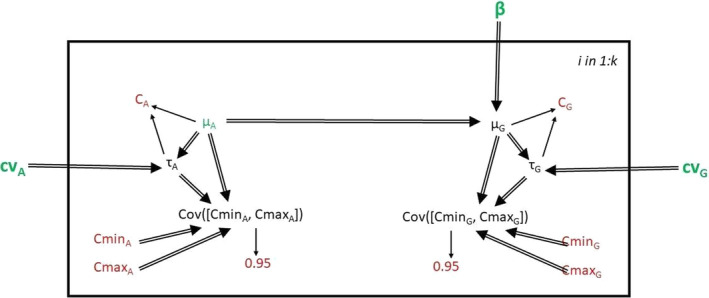
A schematic diagram illustrating the Bayesian model used to estimate the multiplicative conversion factor needed to transform a ground count into an equivalent aerial count. The model estimates the posterior distribution of the conversion factor, β. Data are represented in red; root nodes of the model are shown in green. cv_A_ and cv_G_ are the coefficients of variation associated with the aerial and ground census methods respectively. The model is applied separately to each of the 5 classes of animals defined above.

## Data Availability

Data and R code are available on Zenodo at https://doi.org/10.5281/zenodo.10687543. Due to restricted access, some data are not directly available but they can be provided upon request, subject to a confidentiality clause.

## APPENDIX A

### A.1 BUGS code for trends

model {  # precision is derived from the Confidence Intervals provided in entry for (i in 1:k) { sd[i] <- (h[i]-l[i])/3.93 # normal distribution assumed prec[i] <- pow(sd[i],-2) } lability <- 100 # mild constraint of resemblance between successive r (intrinsic rate of increase) minN1 <- c[1]/2 maxN1 <- c[1]*2 # Priors and constraints N[1] ~ dunif(minN1, maxN1) # initial population size bounded between half and two times the count  # Likelihood # State process logN[1]<-log(N[1]) rcand[1] ~ dnorm(0, 1) # candidate rate of increase r[1] <- min(rcand[1], rmax) # true rate cannot exceed rmax logN[2] <- logN[1]+(t[2]-t[1])*r[1] # r is per unit of time, hence the (t[2]-t[1]) factor to account for the interval duration N[2] <- exp(logN[2]) for (i in 2:(k-1)){ rcand[i] ~ dnorm(r[i-1], lability) # conditions in year i should roughly resemble those in year i-1 r[i] <- min(rcand[i], rmax) # but rate of increase cannot exceed rmax logN[i+1] <- logN[i]+(t[i+1]-t[i])*r[i] # r is per unit of time, hence the (t[i+1]-t[i]) factor to account for the interval duration N[i+1] <- exp(logN[i+1]) } # Observation process for (i in 1:k) { c[i] ~ dnorm(N[i], prec[i]) # observed count is distributed around the true count with the appropriate precision for the date }  # other interesting quantities meanr <- mean(r[]) sdr <- sd(r[]) }

### A.2 Bugs code for deriving conversion factors

The basic data (at our disposal) for deriving conversion factors are pairs of counts carried out in parallel on the same species and at the same time (pair i), one from the air C_Ai_, and one from the ground C_Gi_, along with their associated 95% confidence intervals: C_minAi_ et C_maxAi_, C_minGi_ et C_maxGi_. The pairs of counts for each of the five classes of species retained in section 2.1 are analyzed together in order to estimate the conversion factor for each class.

The estimating model, represented diagrammatically in Figure A1, assumes that the actual count, denoted by C, follows a normal distribution centered on the unknown expected count, μ. Specifically,

C_Ai_ ~ *N* (μ_A,i_, τ_A,i_) where τ_A,i_ = (μ_A,i_ cv_A_)^−2^ is the precision following the convention of BUGS languages

$$C_{\mathrm{Gi}}\sim N\;\left(\mu_{G,i},\tau_{G,i}\right)$$

The model ensures that the count distribution covers 95% of the interval [Cmin_A,i_, Cmax_A,i_] or [Cmin_G,i_, Cmax_G,i_].

The coefficients of variation, cv_A_ and cv_G_, are specific to the field method but independent of the population size, meaning the error on a count is proportional to the population size.

A key element of the model is that the expected aerial and ground counts are proportionally related within a class:

$$\mu_{A,i}=\beta \;\mu_{G,i}$$

Here, β is the class conversion factor, the multiplicative factor applied to a ground count to obtain the equivalent aerial count.

The model is implemented in a Bayesian framework where the root nodes β, cv_A_, cv_G_, and the μ_A,i_ are given the following priors:

$$\mu_{A,i}\sim N\;\left(C_{\mathrm{Ai}},0.01\right)$$

$$\beta \sim N\;\left(1,0.01\right)$$

$${\mathrm{cv}}_{A}\sim \text{Unif}\left(0,100\right)$$

$${\mathrm{cv}}_{G}\sim \text{Unif}\left(0,100\right)$$

We used the program JAGS (Plummer, 2003) called from R (v4.2.3; R Core Team, 2023) with package R2jags (Su & Yajima, 2021). The actual code follows: # count series method m: c[m,i] # count upper boundary: cmax[m,i] # count lower boundary: cmin[m,i]data { # initializations of level of Confidence Intervals  for (m in 1:2) { for (i in 1: k) { ICcoverage[m,i] <- 0.95 ccopy[m,i] <- c[m,i] } }}model { # Priors and constraints # assuming proportional counts for methods 1 and 2 (i.e. a fixed percentage of method 2 relative to method 1) ratio2over1 ~ dnorm(1,0.01) # expected count using method 1# weak prior loosely based on corresponding count for (i in 1:k) { muc[1,i] ~ dnorm(ccopy[1,i],0.01) # expected mean of count using method 1 } # we assume a constant coefficient of variation for both methods cv[1] ~ dunif(0,100) # coefficient of variation of counts in method 1 cv[2] ~ dunif(0,100) # coefficient of variation of counts in method 2 # likelihood for (i in 1:k) { muc[2,i] <- muc[1,i]*ratio2over1 for (m in 1:2) { tau[m,i] <- pow(muc[m,i]*cv[m],-2)# the actual count is assumed to follow a normal distribution centered around the expected count muc[m,i] c[m,i] ~ dnorm(muc[m,i],tau[m,i])# coverage of confidence interval deltaprob[m,i] <- pnorm(cmax[m,i],muc[m,i],tau[m,i])-pnorm(cmin[m,i],muc[m,i],tau[m,i])# As the observed node must be stochastic, the calculated coverage, deltaprob, is assumed to be normally distributed # around its nominal 95% value, but with very high precision (10000) ICcoverage[m,i]~ dnorm(deltaprob[m,i],10000)}}}

## References

[ece370193-bib-0001] Agriculture Ministers' Conference . (2010). History of rinderpest eradication from Africa, impact, lessons learnt and way forward (p. 10). African Union, InterAfrican Bureau for Animal Resources. https://www.au‐ibar.org

[ece370193-bib-0002] Amano, T. , Okamura, H. , Carrizo, S. F. , & Sutherland, W. J. (2012). Hierarchical models for smoothed population indices: The importance of considering variations in trends of count data among sites. Ecological Indicators, 13, 243–252. 10.1016/j.ecolind.2011.06.008

[ece370193-bib-0003] Arciszewski, T. J. , Roberts, D. R. , Mahaffey, A. , & Hazewinkel, R. R. O. (2023). Distinguishing between research and monitoring programs in environmental science and management. Journal of Environmental Studies and Sciences, 13, 674–681. 10.1007/s13412-023-00859-0

[ece370193-bib-0004] Barnes, M. D. , Craigie, I. D. , Harrison, L. B. , Geldmann, J. , Collen, B. , Whitmee, S. , Balmford, A. , Burgess, N. D. , Brooks, T. , Hockings, M. , & Woodley, S. (2016). Wildlife population trends in protected areas predicted by national socio‐economic metrics and body size. Nature Communications, 7, 12747. 10.1038/ncomms12747 PMC5025815 27582180

[ece370193-bib-0005] Bart, J. , Collins, B. , & Morrison, R. I. G. (2003). Estimating population trends with a linear model. The Condor, 105, 367–372.

[ece370193-bib-0006] Blum, M. E. , Buderman, F. E. , Bennett, J. R. , Stewart, K. M. , Cox, M. , & Williams, P. J. (2024). Comparing contemporary models to traditional indices to estimate abundance of desert bighorn sheep. Journal of Wildlife Management, 88, e22517. 10.1002/jwmg.22517

[ece370193-bib-0007] Borchers, D. L. , Buckland, S. T. , & Zucchini, W. (2002). Estimating animal abundance: Closed populations (p. 314). Springer Science & Business Media. 10.1007/978-1-4471-3708-5

[ece370193-bib-0008] Bouché, P. , Renaud, P.‐C. , Lejeune, P. , Vermeulen, C. , Froment, J.‐M. , Bangara, A. , Fiongai, O. , Abdoulaye, A. , Abakar, R. , & Fay, M. (2009). Has the final countdown to wildlife extinction in northern Central African Republic begun? African Journal of Ecology, 48, 994–1003.

[ece370193-bib-0009] Boyd, C. , & Punt, A. E. (2021). Shifting trends: Detecting changes in cetacean population dynamics in shifting habitat. PLoS One, 16, e0251522. 10.1371/journal.pone.0251522 34014942 PMC8136736

[ece370193-bib-0010] Bristow, K. D. , Clement, M. J. , Crabb, M. L. , Harding, L. E. , & Rubin, E. S. (2019). Comparison of aerial survey methods for elk in Arizona. Wildlife Society Bulletin, 43, 77–92. 10.1002/wsb.940

[ece370193-bib-0011] Burton, A. C. (2012). Critical evaluation of a long‐term, locally‐based wildlife monitoring program in West Africa. Biodiversity and Conservation, 21, 3079–3094. 10.1007/s10531-012-0355-6

[ece370193-bib-0012] Casajus, N. , & Pradel, R. (2023). popbayes: Bayesian model to estimate population trends from counts series . R package version 1.2.0. https://frbcesab.github.io/popbayes/

[ece370193-bib-0013] Caughlan, L. , & Oakley, K. L. (2001). Cost considerations for long‐term ecological monitoring. Ecological Indicators, 1, 123–134.

[ece370193-bib-0014] Ceballos, G. , Ehrlich, P. R. , Barnosky, A. D. , García, A. , Pringle, R. M. , & Palmer, T. M. (2015). Accelerated modern human‐induced species losses: Entering the sixth mass extinction. Science Advances, 1(5), e1400253. 10.1126/sciadv.1400253 26601195 PMC4640606

[ece370193-bib-0015] Child, G. , Robbel, H. , & Hepburn, C. P. (1972). Observations on the biology of tsessebe, *Damaliscus lunatus lunatus*, in northern Botswana. Mammalia, 36, 342–388. 10.1515/mamm.1972.36.3.342

[ece370193-bib-0067] Cornélis, D. (2000). Analyse du monitoring écologique et cynégétique des populations des principaux ongulés au Ranch de Gibier de Nazinga (Burkina Faso) (p. 113). Fac. Universitaire des Sc. Agro Gembloux.

[ece370193-bib-0066] Cornélis, D. , Melletti, M. , Korte, L. , Ryan, S. J. , Mirabile, M. , Prin, T. , Prins, H. H. (2014). African buffalo Syncerus caffer (Sparrman, 1779). In Ecology, evolution and behaviour of wild cattle: Implications for conservation (pp. 326–372). Cambridge University Press.

[ece370193-bib-0016] Craigie, I. D. , Baillie, J. E. M. , Balmford, A. , Carbone, C. , Collen, B. , Green, R. E. , & Hutton, J. M. (2010). Large mammal population declines in Africa's protected areas. Biological Conservation, 143(9), 2221–2228. 10.1016/j.biocon.2010.06.007

[ece370193-bib-0017] Dennis, B. , Ponciano, J. M. , Lele, S. R. , Taper, M. L. , & Staples, D. F. (2006). Estimating density dependence, process noise, and observation error. Ecological Monographs, 76, 323–341.

[ece370193-bib-0018] Drescher, M. , Perera, A. H. , Johnson, C. J. , Buse, L. J. , Drew, C. A. , & Burgman, M. A. (2013). Toward rigorous use of expert knowledge in ecological research. Ecosphere, 4, 1–26. 10.1890/ES12-00415.1

[ece370193-bib-0019] East, R. (1999). African antelope database 1998 (p. 434). IUCN/SSC Antelope Specialist Group.

[ece370193-bib-0020] Eikelboom, J. A. J. , Wind, J. , van de Ven, E. , Kenana, L. M. , Schroder, B. , de Knegt, H. J. , van Langevelde, F. , & Prins, H. H. T. (2019). Improving the precision and accuracy of animal population estimates with aerial image object detection. Methods in Ecology and Evolution, 10(11), 1875–1887. 10.1111/2041-210X.13277

[ece370193-bib-0021] Fewster, R. M. , Buckland, S. T. , Siriwardena, G. M. , Baillie, S. R. , & Wilson, J. D. (2000). Analysis of population trends for farmland birds using generalized additive models. Ecology, 81, 1970–1984. 10.1890/0012-9658(2000)081[1970:AOPTFF]2.0.CO;2

[ece370193-bib-0022] Fisher, R. A. (1930). The genetical theory of natural selection (p. 356). Clarendon Press.

[ece370193-bib-0068] Foley, C. A. H. , & Faust, L. J. (2010). Rapid population growth in an elephant Loxodonta africana population recovering from poaching in Tarangire National Park, Tanzania. Oryx, 44(2), 205–212. 10.1017/S0030605309990706

[ece370193-bib-0023] Forney, K. A. , Moore, J. E. , Barlow, J. , Carretta, J. V. , & Benson, S. R. (2021). A multidecadal Bayesian trend analysis of harbor porpoise (*Phocoena phocoena*) populations off California relative to past fishery bycatch. Marine Mammal Science, 37, 546–560. 10.1111/mms.12764

[ece370193-bib-0024] Frankel, A. S. , Gabriele, C. M. , Yin, S. , & Rickards, S. H. (2022). Humpback whale abundance in Hawai'i: Temporal trends and response to climatic drivers. Marine Mammal Science, 38, 118–138. 10.1111/mms.12856

[ece370193-bib-0025] Gaidet‐Drapier, N. , Fritz, H. , Bourgarel, M. , Renaud, P.‐C. , Poilecot, P. , Chardonnet, P. , Coid, C. , Poulet, D. , & Le Bel, S. (2006). Cost and efficiency of large mammal census techniques: Comparison of methods for a participatory approach in a communal area, Zimbabwe. Biodiversity and Conservation, 15, 735–754. 10.1007/s10531-004-1063-7

[ece370193-bib-0026] Greene, K. , Bell, D. , Kioko, J. , & Kiffner, C. (2017). Performance of ground‐based and aerial survey methods for monitoring wildlife assemblages in a conservation area of northern Tanzania. European Journal of Wildlife Research, 63, 1–13. 10.1007/s10344-017-1133-2

[ece370193-bib-0027] Hammond, P. S. , Francis, T. B. , Heinemann, D. , Long, K. J. , Moore, J. E. , Punt, A. E. , Reeves, R. R. , Sepúlveda, M. , Sigurðsson, G. M. , Siple, M. C. , Víkingsson, G. , Wade, P. R. , Williams, R. , & Zerbini, A. N. (2021). Estimating the abundance of marine mammal populations. Frontiers in Marine Science, 8, 735770. 10.3389/fmars.2021.735770

[ece370193-bib-0028] Hennig, J. D. , & Schoenecker, K. A. (2023). Comparing methods to estimate feral burro abundance. Wildlife Society Bulletin, 47(4), e1495. 10.1002/wsb.1495

[ece370193-bib-0029] Hone, J. (1999). On rate of increase (r): Patterns of variation in Australian mammals and the implications for wildlife management. Journal of Applied Ecology, 36, 709–718. 10.1046/j.1365-2664.1999.00439.x

[ece370193-bib-0030] Hone, J. , Duncan, R. P. , & Forsyth, D. M. (2010). Estimates of maximum annual population growth rates (r_m_) of mammals and their application in wildlife management. Journal of Applied Ecology, 47(3), 507–514. 10.1111/j.1365-2664.2010.01812.x

[ece370193-bib-0031] Hostetler, J. A. , & Chandler, R. B. (2015). Improved state‐space models for inference about spatial and temporal variation in abundance from count data. Ecology, 96, 1713–1723.

[ece370193-bib-0032] Humbert, J.‐Y. , Scott Mills, L. , Horne, J. S. , & Dennis, B. (2009). A better way to estimate population trends. Oikos, 118, 1940–1946. 10.1111/j.1600-0706.2009.17839.x

[ece370193-bib-0033] Infantes, E. , Carroll, D. , Silva, W. T. A. F. , Härkönen, T. , Edwards, S. V. , & Harding, K. C. (2022). An automated work‐flow for pinniped surveys: A new tool for monitoring population dynamics. Frontiers in Ecology and Evolution, 10, 905309. 10.3389/fevo.2022.905309

[ece370193-bib-0034] Jachmann, H. (2002). Comparison of aerial counts with ground counts for large African herbivores. Journal of Applied Ecology, 39, 841–852.

[ece370193-bib-0035] Kidwai, Z. , Jimenez, J. , Louw, C. J. , Nel, H. P. , & Marshal, J. P. (2019). Using N‐mixture models to estimate abundance and temporal trends of black rhinoceros (*Diceros bicornis* L.) populations from aerial counts. Global Ecology and Conservation, 19, e00687. 10.1016/j.gecco.2019.e00687

[ece370193-bib-0036] Kingdon, J. , & Hoffmann, M. (2013). The mammals of Africa. Volume VI: Pigs, hippopotamuses, chevrotain, giraffes, deer and bovids (p. 704). Bloomsbury Publishing.

[ece370193-bib-0037] Kouba, V. (2013). Rinderpest global eradication management. Agricultura Tropica et Subtropica, 46(2), 35–42.

[ece370193-bib-0038] Krebs, C. J. (2006). Mammals. In W. J. Sutherland (Ed.), Ecological census techniques: A handbook (pp. 351–369). Cambridge University Press.

[ece370193-bib-0039] Kuhnert, P. M. , Martin, T. G. , & Griffiths, S. P. (2010). A guide to eliciting and using expert knowledge in Bayesian ecological models. Ecology Letters, 13, 900–914. 10.1111/j.1461-0248.2010.01477.x 20497209

[ece370193-bib-0040] Mazzetta, C. , Brooks, S. , & Freeman, S. N. (2007). On smoothing trends in population index modeling. Biometrics, 63, 1007–1014. 10.1111/j.1541-0420.2007.00820.x 17501945

[ece370193-bib-0041] Plummer, M. (2003). JAGS: A program for analysis of Bayesian graphical models using Gibbs sampling. Proceedings of the 3rd International Workshop on Distributed Statistical Computing (DSC 2003), Vienna, Austria, 1–10.

[ece370193-bib-0042] R Core Team . (2023). R: A language and environment for statistical computing. R Foundation for Statistical Computing. https://www.R‐project.org/

[ece370193-bib-0043] Redfern, J. , Viljoen, P. , Botha, J. , & Getz, W. (2002). Biases in estimating population size from an aerial census: A case study in the Kruger National Park, South Africa. South African Journal of Science, 98(9), 455–461.

[ece370193-bib-0044] Renaud, P. C. (2005). Recensement aérien de la faune dans les préfectures de la région Nord de la République Centrafricaine (p. 53). Université de Bangui. 10.13140/2.1.2502.0800

[ece370193-bib-0045] Renaud, P. C. , Gueye, M. B. , Hejcmanová, P. , Antonínová, M. , & Samb, M. (2006). Inventaire aérien et terrestre de la faune et relevé des pressions au Parc National du Niokolo Koba (p. 57). Ministère de l'Environnement et de la Protection de la Nature du Sénégal and African Parks Foundation.

[ece370193-bib-0046] Ridpath, M. G. , Begg, R. J. , Dudzinski, M. L. , Forbes, M. A. , & Graham, A. (1983). Counting the same populations of large tropical mammals from the ground and from the air. Australian Wildlife Research, 10, 487–498.

[ece370193-bib-0047] Sachs, R. (1967). Liveweights and body measurements of Serengeti game animals. African Journal of Ecology, 5, 24–36. 10.1111/j.1365-2028.1967.tb00758.x

[ece370193-bib-0048] Sands, J. P. , & Pope, M. D. (2010). A survey of galliform monitoring programs and methods in the United States and Canada. Wildlife Biology, 16, 342–356. 10.2981/09-066

[ece370193-bib-0049] Schaub, M. , & Kery, M. (2021). Integrated population models: Theory and ecological applications with R and JAGS (p. 638). Academic Press.

[ece370193-bib-0050] Scholte, P. , Pays, O. , Adam, S. , Chardonnet, B. , Fritz, H. , Mamang, J.‐B. , Prins, H. H. T. , Renaud, P.‐C. , Tadjo, P. , & Moritz, M. (2022). Conservation overstretch and long‐term decline of wildlife and tourism in the central African savannas. Conservation Biology, 36, e13860. 34766386 10.1111/cobi.13860

[ece370193-bib-0051] Shamon, H. , Cosby, O. G. , Andersen, C. L. , Augare, H. , BearCub Stiffarm, J. , Bresnan, C. E. , Brock, B. L. , Carlson, E. , Deichmann, J. L. , Epps, A. , Guernsey, N. , Hartway, C. , Jørgensen, D. , Kipp, W. , Kinsey, D. , Komatsu, K. J. , Kunkel, K. , Magnan, R. , Martin, J. M. , … Akre, T. S. (2022). The potential of bison restoration as an ecological approach to future tribal food sovereignty on the northern Great Plains. Frontiers in Ecology and Evolution, 10, 826282. 10.3389/fevo.2022.826282

[ece370193-bib-0052] Sibly, R. M. , & Hone, J. (2002). Population growth rate and its determinants: An overview. Philosophical Transactions of the Royal Society B, 357, 1153–1170. 10.1098/rstb.2002.1117 PMC1693026 12396508

[ece370193-bib-0053] Sinclair, A. R. E. (1996). Mammal populations: Fluctuation, regulation, life history theory, and their implications for conservation. In R. B. Floyd , A. W. Sheppard , & P. J. De Barro (Eds.), Frontiers of population ecology (pp. 101–128). CSIRO.

[ece370193-bib-0054] Sinclair, A. R. E. (2003). Mammal population regulation, keystone processes and ecosystem dynamics. Philosophical Transactions of the Royal Society of London. Series B, Biological Sciences, 358, 1729–1740. 10.1098/rstb.2003.1359 14561329 PMC1693264

[ece370193-bib-0055] Stalmans, M. E. , Massad, T. J. , Peel, M. J. S. , Tarnita, C. E. , & Pringle, R. M. (2019). War‐induced collapse and asymmetric recovery of large‐mammal populations in Gorongosa National Park, Mozambique. PLoS One, 14(3), e0212864. 10.1371/journal.pone.0212864 30865663 PMC6415879

[ece370193-bib-0056] Su, Y. S. , & Yajima, M. (2021). R2jags: Using R to run ‘JAGS’ . R package version 0.7–1. https://CRAN.R‐project.org/package=R2jags

[ece370193-bib-0057] Suraud, J. P. , Fennessy, J. , Bonnaud, E. , Issa, A. M. , Fritz, H. , & Gaillard, J.‐M. (2012). Higher than expected growth rate of the endangered west African giraffe *Giraffa camelopardalis peralta*: A successful human–wildlife cohabitation. Oryx, 46(4), 577–583. 10.1017/S0030605311000639

[ece370193-bib-0058] Sutherland, W. J. (2006). Ecological census techniques: A handbook (p. 450). Cambridge University Press.

[ece370193-bib-0059] ter Braak, C. J. F. , van Strien, A. J. , Meijer, R. , & Verstrael, T. J. (1994). Analysis of monitoring data with many missing values: Which method? In E. J. M. Hagemeijer & T. J. Verstrael (Eds.), Bird numbers 1992. Distribution, monitoring and ecological aspects. Proceedings 12th international conference of IBCC and EOAC, Noordwijkerhout, The Netherlands (pp. 663–673). Statistics Netherlands, SOVON, Beek‐Ubbergen.

[ece370193-bib-0060] Terletzky, P. A. , & Koons, D. N. (2016). Estimating ungulate abundance while accounting for multiple sources of observation error. Wildlife Society Bulletin, 40, 525–536. 10.1002/wsb.672

[ece370193-bib-0061] Tolimieri, N. , Holmes, E. E. , Williams, G. D. , Pacunski, R. , & Lowry, D. (2017). Population assessment using multivariate time‐series analysis: A case study of rockfishes in Puget Sound. Ecology and Evolution, 7(8), 2846–2860. 10.1002/ece3.2901 28428874 PMC5395462

[ece370193-bib-0062] Tounkara, K. , Nwankpa, N. , & Bodjo, S.‐C. (2017). Rinderpest experience. Revue Scientifique et Technique/Office International des Épizooties, 36(2), 569–578. 10.20506/rst.36.2.2675 30152462

[ece370193-bib-0063] Van Hensbergen, H. J. , & White, G. C. (1995). Review of methods for monitoring vertebrate population parameters. In J. A. Bissonette & P. R. Krausman (Eds.), Integrating people and wildlife for a sustainable future (pp. 489–508). Wildlife Society. 10.13140/RG.2.1.4915.3681

[ece370193-bib-0064] Wood, S. N. (2017). Generalized additive models: An introduction with R, second edition (p. 496). Chapman & Hall/CRC.

[ece370193-bib-0065] Yeiser, J. M. , Morgan, J. J. , Baxley, D. L. , Chandler, R. B. , & Martin, J. A. (2018). Private land conservation has landscape‐scale benefits for wildlife in agroecosystems. Journal of Applied Ecology, 55, 1930–1939. 10.1111/1365-2664.13136

